# The Foreign Journals: Midwifery, and Diseases of Women and Children

**Published:** 1842-04

**Authors:** 


### MIDWIFERY, AND DISEASES OF WOMEN AND CHILDREN.

*Case of Chronic Hydrocephalus occurring in a Boy nine Years of Age.* By MM. BARTHEZ and RILLIET.

THE subject of this observation was a healthy child, and remarkably intelligent and quick of apprehension. At the age of seven years and half he had a severe fall, after which he frequently complained of pains in the back of his neck. It was not, however, until six months afterwards—in September, 1839—

that any more serious symptoms became apparent. At the end of September he could scarcely see to read, and when writing would frequently mistake one line for another. About the middle of October his gait became tottering, but no enlargement of the head was perceived before the middle of November. In January, 1840, his sight was almost entirely gone, and in March he became completely blind, and was forced to keep his bed by inability to walk. In April he began to pass his urine and feces involuntarily, and for three weeks had copious vomiting after every meal. The size of the head had gone on increasing ever since November, 1839; and in May, 1840, there came on a constant tremulous motion of the upper extremities. Notwithstanding the progress of the disease, the digestive and respiratory functions continued natural and the intellectual powers perfect, the patient recognizing persons by their voices. Leeches, setons, purgatives, and various other remedies had been employed from October, 1839 to August, 1840, when the child was admitted into the Hôpital des Enfans.

The head at that time measured 60 centimetres, about 23¾ inches, in circumference; 41 centimetres, or 16 inches, from the root of the nose to the occipital protuberance; and 39 centimetres, or 15 inches, from one ear to the other. The child lay on his back; his countenance was placid; the pulse 108, and regular. When spoken to, he answered slowly, but correctly, though not without hesitation. If spoken to suddenly, or at all excited, his upper extremities are seized with tremor; occasional rigidity of the fore-arm, and contractions of the thumb into the palm were likewise observed. He retained power, however, over the upper extremities, and could clench the hand firmly. He could move his legs, though unable to stand, or even to sit firmly. There was no pain along the spinal column.

Such was the condition of the child on the 7th of August, and this state continued up to the 20th, when he was suddenly seized with general convulsions, which lasted for an hour, when an interval of an hour occurred, after which they returned with increased intensity. At nine, p. m., the features were drawn to the left side, the fore-arms were rigid and bent, the fingers clenched, and the thumbs drawn into the palms, while the whole trunk was as stiff as a bar of iron. Sensibility was now completely abolished, and the child continued in this state, interrupted only by occasional convulsions, till eleven, p. m., when he died, nine hours after the first attack.

The bones of the cranium were found to be very thin; the brain was large, its convolutions were expanded, and its ventricles contained three fourths of a litre, 1¾ pint of limpid serum. The cerebellum was estimated to be a fifth larger than natural; the whole of the interior of its right lobe was in a state of softening, which condition extended as far as the walls of the fourth ventricle, and the *tubercular quadrigemina.* In the midst of this softened tissue, which was white in some parts, and of a yellowish red in others, there were four or five tumours, the smallest of which was of the size of a lentil, the largest as big as a nut. Some of them were near the surface, others deep in the substance of the cerebellum. They were irregular in form, but slightly rounded, consisting of a dense elastic substance, of a blueish or dead white colour, and resembling the inter-articular fibro-cartilages. They adhered firmly to the softened cerebral substance, and one of them which was in contact with the dura mater could hardly be detached from it. The spinal cord presented a precisely similar lesion. A little below the cervical expansion of the cord, there was a patch of morbid structure, fifteen millimetres (half an inch) long, ten (one third of an inch) wide, and six (one fourth of an inch) thick. Externally it was firmly connected with the dura mater; internally it adhered at its centre to the medullary substance, from which, however, it was possible to separate it completely. The spinal cord in the situation of this tumour was twice as large as either above or below it; its substance was slightly red and softened.

The writers propose three hypotheses as to the origin of these tumours; either that they result from a degeneration of the tissue of the pia mater; or from hypertrophy, with induration of the cerebral substance itself; or from an extravasation of blood either into the cerebral substance or the pia mater. They incline to the third supposition, and conceive that the alterations which a fibrinous clot may undergo would produce tumours similar to those described above. In confirmation of this view, they detail a case in which, in a child aged seven years, a tumour of this character was found in the immediate vicinity of several apoplectic cells.

*Archives Générales de Médecine. Janvier,* 1842.

---

*Account of a Case of Extra-Uterine Pregnancy in which Gastrotomy was successfully performed.* By MM. Mathieu and Bressolles, of the Hospital of La Charité sur Loire.

This case has given rise to disputes between MM. Mathieu and Bressolles, as to the share which each had in the performance of the operation. But, leaving these matters, and the opinions which M. Mathieu has expressed, apparently on insufficient grounds with respect to the membranes of the fœtus, the following particulars of the case may be of interest.

The woman was thirty-eight years old, and had given birth to three children by her first husband. After his death she married again, but three years had passed without any sign of pregnancy, when at the commencement of January, 1835, the flow of the menses became extremely scanty, and on the following month ceased altogether. The woman experienced other symptoms of pregnancy, the abdomen enlarged, and about the middle of June the movements of the child became perceptible, and continued at intervals till the beginning of November. Pains like those of labour then came on, the patient experienced a sensation as of some violent tremulous motion of the fœtus, followed by three smart shocks, after which all movements ceased, and she supposed that the child had died.

During the three weeks preceding this occurrence the patient had suffered severe pains, which led her to think that labour was coming on. Several midwives and some medical men were sent for, who coincided in the opinion that the case was one of extra-uterine pregnancy; and the latter, according to M. Bressolles, suggested the performance of gastrotomy to which, however, the husband would not consent.

During the next six months, the patient's condition grew more and more intolerable. She had frequent attacks of colic; vomiting, usually of a bilious fluid, and obstinate constipation, which could be relieved only by frequently repeated injections. She could obtain no rest, was subject to a constant fever, fainted often, was much distressed by the sensation of something rolling about in her abdomen; and latterly, ascites and œdema of the extremities had supervened. Some of the more alarming of these symptoms subsided, but the patient still suffered much pain, and became daily more and more emaciated, which circumstances induced her to apply at the hospital.

At the time of her admission the abdomen was so much distended by fluid that it was necessary to perform paracentesis, in order to ascertain the patient's real condition. It was then placed beyond all doubt that a fœtus was contained in the abdomen, that its head was situated in the hypogastric region, while the feet could be felt at the right side. Besides this a tumour was felt in the epigastrium which pushed the xiphoid cartilage forward, but the nature of this growth it was not possible to determine.

On the 27th of August, 1836, fifteen days after the patient's admission into the hospital, M. Mathieu proceeded to the operation, by making an incision from the umbilicus to the pubes in order to remove the child, which was about nineteen inches long, and had undergone no change, except the separation of a little hair from the temples. The hand was next introduced to remove the placenta, which adhered to the right iliac fossa, but it was not found possible to abstract more than a few portions of it. The incision was now enlarged upwards, and M. Bressolles, who had removed the placenta, detached the epigastric tumour by means of a pair of curved scissors. It was found to be attached to the diaphragm close to its insertion into the sternum; it was of an oval form, as large as the head of a fœtus at the sixth month, and quite free from all connexion with the stomach. Its parietes were cartilaginous; it contained steatomatous matter and hair, and a small portion of bone, which M. Mathieu describes as resembling a fœtal bone, though the existence of any such similarity is totally denied by M. Bressolles.

The edges of the wound were brought together by suture, and a pledget of lint placed at its lower angle to facilitate the escape of any purulent matters from the abdomen. The patient recovered without a single bad symptom; the very copious purulent secretion from the abdominal cavity was the most remarkable feature in her convalescence. When she left the hospital a small fistulous opening existed at the lower part of the wound, but when the patient was seen again on June 20, 1837, this had closed and was succeeded by another close under the umbilicus. At this time her health was good; she had gained flesh, and could rest in any posture, though she experienced a dull pain in the right iliac region whenever she stood or walked. There was no hardness in the epigastric region, and the abdomen generally was soft.

Menstruation had occurred for the first time after the operation on May 1, and had reappeared on the 1st of June.

MM. Capuron and Gerdy, in their report on the case to the Academy of Medicine, have collected thirty-three other observations of extra-uterine pregnancy, to which we refer the reader. We cannot conceal our regret that the details of so interesting an observation should be disfigured by the squabbles of men who might have been expected to carry into the exercise of their profession higher motives than those of a vulgar and selfish appetite for distinction.

*Bulletin de l'Académie Royale de Médecine. Sept.* 30, 1841.

and *Annales de la Chirurgie. Sept.* 1841.

---

*On Inflammation of the Mucous Follicles of the Vulva, and its Treatment.*
By M. A. Robert.

This affection frequently accompanies blenorrhagia, or occurs in women who suffer from sub-acute inflammations of the mucous membrane of the uterus, or is induced by pregnancy or abortion, or appears as a consequence of derangements of the menses. The chief seat of the inflammation in these cases is to be found in those two large mucous follicles situated at the entrance of the vagina, and known by the name of the glands of Duverney or of Bartholinus.

Its chief symptoms are a darting pain and distressing pruritus at the vulva, aggravated by all the patient's movements, and especially by sexual intercourse. Considerable redness is observable at the entrance of the vagina; sometimes especially obvious at the two points which correspond to the openings of the mucous follicles, from which pus can usually be squeezed by slight pressure.

The nature of the affection is frequently mistaken by practitioners, who sometimes attribute the itching to the irritation of the mucous membrane of the vagina from habitual leucorrhea; others conceive it to be a form of porrigo, or look on it as the consequence of syphilitic infection. Nothing, however, is easier than its cure when the real nature of the affection has been ascertained. It consists in introducing one of Anel's stylets into the follicle, then laying its cavity open by means of a pair of probe-pointed scissors, and cauterizing its internal surface with nitrate of silver. The operation, though painful, is never dangerous, and is always succeeded by a complete cure, provided the whole of the follicle has been destroyed, but when this has not been effected, a repetition of the operation may become necessary. Some cases are detailed in illustration of the benefits of this procedure, where the patients had suffered from the affection for years, and undergone a variety of treatment without effect.

*Bulletin Général de Thérapeutique. Sept.* 30, 1841.

*On the Pulse of Infants at the Breast.* By Professor TROUSSEAU.

THE following are the results of the observations of M. Trousseau:

In children in the first weeks of life the pulse varies, according to Billard and Trousseau, from 78 to 150; according to M. Valleix, from 76 to 104 only; at a more advanced age according to the observations of M. Trousseau,

| | | |
|---|---|---|
| In the second half of the first month from | 120 to | 164 |
| From one to two months . . . . | 96 | 132 |
| ,, two to six . . . . . . . . | 100 | 162 |
| ,, six to twelve . . . . . | 100 | 160 |
| ,, twelve to twenty-one . . . . | 96 | 140 |

It is a little more frequent in girls than in boys after the first two months. In children from fifteen days to six months the pulse is 180 when awake, 121 during sleep. In children from six to twenty-one months 128 when awake, 112 during sleep. Thus when in a sleeping child we find the pulse at 140, and in a wakeful 150 or 155, we may generally suspect that fever is present.

*L'Examinateur Médical. Août* 29, 1841.

---

*Extirpation of a Diseased Ovary.* By Dr. STILLING, of Cassell.

THE patient, an unmarried female, 22 years old, had always enjoyed good health, with the exception of a tumour which had been growing for three years and a half in the right side of the abdomen. This tumour occasioned her so much inconvenience, that she determined to undergo any operation which might be necessary for its removal.

April 30th, the patient being placed on the operating table at 8 a.m., an incision was made four inches in length, from the umbilicus downwards in the direction of the linea alba. The tumour was now punctured, and gave exit to a tenacious, gelatinous matter of a yellow colour. It was found that the tumour was divided by septa, so that it was necessary to puncture several cysts before its size was reduced sufficiently to allow of the completion of the operation.

The incision was somewhat enlarged by cutting for two inches above the navel, and the tumour was then drawn forwards by hooks. It was about the size of a man's head, and was formed by the right ovary, which was connected with the uterus by the broad ligament, and by a bundle of vessels about the thickness of the finger. A strong, double, silk ligature, was placed round this pedicle by means of a needle, and its ends were firmly tied on either side. The pedicle was now cut through; and the tumour, which was not adherent to any other organ, was removed without the loss of any blood. The ovary weighed six pounds, and the contents removed from it during the operation were found to weigh eighteen pounds.

Four sutures were applied, a bandage passed round the abdomen, and the patient removed to bed, the operation and dressing of the wound having occupied three quarters of an hour.

During the operation no untoward accident happened, and the patient's condition was very satisfactory for twenty-four hours afterwards. Symptoms of peritonitis then came on, which were checked by venesection, but the patient continued restless and feverish during the second night after the operation, and was much troubled by retching and occasional vomiting. Attempts were made to induce action of the bowels by purgative pills, but their only effect was to induce vomiting. At midnight about six ounces of blood escaped through the bandage, and for a time the pulse became extremely frequent. On May 3, the patient was evidently much worse, the pulse was almost imperceptible, vomiting incessant; she complained of singing in the ears, and of great oppression at the chest, and the same day at half-past eleven she died.

A considerable quantity of blood flowed from the wound in carrying the body to the room where the post-mortem examination was made. A small quantity of coagulated blood was found in the right iliac region, and appeared to have escaped from the pedicle, to the cut surface of which coagulated blood was adhering. It seems therefore that the ligature, though tight, was insufficient to prevent hemorrhage. The peritoneal coat of the anterior part of the uterus was inflamed and covered with lymph. The peritoneum around the wound seemed slightly inflamed, but all other parts of the body were perfectly healthy. *Holscher's Hannoversche Annalen.* Heft iii. 1841.

*Injury to the Head of the Child during its passage through the Pelvis in labour.*
By Dr. Schöller, Assistant at the Lying-in-Hospital, Berlin.

A healthy woman, pregnant for the first time, fell in labour on Aug. 23, 1839. The first stage of parturition proceeded extremely slowly, and the passage of the head was evidently retarded by smallness of the pelvis. At the end of three days, and after the employment of venesection and opium, a male child, weighing 6½ pounds, was born. The infant appeared to be in a state of asphyxia, but from this it speedily recovered.

The head was a quarter of an inch smaller than natural in every diameter; a large *caput succedaneum* occupied the right parietal bone, and in the middle of the left parietal bone, and in the neighbourhood of the left temple the skin was abraded and the bone depressed. The skin about these parts became gangrenous, and a red line of demarcation surrounded the mortified structures. The bone beneath likewise died, and a portion as large as a sixpence of the whole thickness of the parietal bone exfoliated, leaving the dura mater exposed. The destruction of the frontal bone was less considerable, and was replaced by granulation, which, on Sept. 13, had likewise advanced so far towards restoring the lost portion of the parietal bone, that the child was dismissed from the hospital. *Medicinische Zeitung. Sept.* 22, 1841.

*Spontaneous Inversion and gradual Replacement of the Uterus.*
By Dr. Borggreve, of Westphalia.

The inversion of the uterus was detected on the third day after the delivery of the patient, a young primipara. It had not occurred during the labour, but at what time or in what manner it took place was not clearly known. The uterus was protruded nearly an inch from the external orifice of the vagina, and its mouth was carried high up. Its form was that of a long flask, for its orifice was completely contracted. Efforts were made to replace it, but they were useless and produced intolerable pain. Ether and opium were therefore administered, anodyne injections were thrown into the vagina, and a sponge was kept in it to prevent the complete descent of the uterus. The patient had frequent syncope, her voice was scarcely audible, her pulse thready, and her countenance deathlike. A fresh attempt at replacement only proved the impossibility of affecting it, for the os uteri was as much closed as ever, and the endeavour produced syncope and convulsions. The urine which had been retained ever since the uterus was inverted, was daily drawn off. Repeated bleedings taking place, the author determined to try to effect an invagination of the uterus so as to diminish its volume, and give a passage to the urine, and perhaps to reinvert it if the peritoneal surfaces had not already become adherent. For this purpose he had a staff eight inches long made, with a knob of the size and form of a hen's egg at one extremity. Then, having with the end of the thumb pressed the fundus of the inverted uterus as far as possible back into the cavity, he introduced the knob of the staff into the depression thus formed, and fixed the other end of it by a moderately tight T bandage. After this the woman had but little pain; in the evening her urine passed freely and all bleeding had ceased. On the sixth day after delivery, and the third after the introduction of the staff, the author withdrew it, and cleansed the vagina. The fundus uteri had already been forced two inches upwards; the knob of the staff was therefore again placed in it, and the rest of it fixed as before. Next morning, after a good night's rest, the uterus was found completely replaced. The os uteri was low down, scarcely an inch and a half from the external orifice, and surrounded the staff. The latter being withdrawn, the patient's strength rapidly increased; in a few days she was completely restored, and several months afterwards, no local disorder could be discerned.

The author mentions as the peculiar advantages of this plan that, by the pressure of the knob of the staff, all the hemorrhage which usually comes from that part of the inverted uterus to which the placenta was attached, is at once checked, and the free passage of the urine is permitted; if there be any spasmodic contraction of the os uteri, it is removed by the permanent pressure on the fundus; and if, as the consequence of inversion of longer standing, the orifice of the uterus is more fixedly contracted, it is dilated, and the complete replacement is by this method permitted to take place naturally and in the most speedy manner possible. *Medicinische Zeitung. Juni* 9, 1841.

*On the Employment of a Ring for retaining the Womb in Cases of complete Prolapsus Uteri.* By Dr. DOMMES, of Hanover.

IN cases where the vagina is too irritable to bear a pessary, or where pessaries have been tried without success, or where extensive and incurable laceration of the perineum exists, Dr. Dommes has resorted with success to the following method:

The patient is seated on a table with the thighs separated; and, after the uterus has been returned, a trocar is thrust through each labium, in the situation of the posterior commissure, from within outwards, and about four lines from its outer edge. Through this wound a leaden wire about four inches long is passed, and its ends are twisted together so as to retain it in its place. The object of this is to prevent the perforations from closing, and, as soon as the swelling of the parts has subsided, and the wound healed, which generally occurs in the course of a few days, a silver ring is substituted for the leaden wire. This ring is about the size of a sixpence, it opens and shuts with a hinge, and the two ends fit into each other, and are kept closed by a little collar which is made perfectly smooth. Dr. Dommes details the case of a person who had a very extensive laceration of the perineum, and a prolapsus uteri before deemed incurable, but who, after employing this ring, married and became pregnant; and he states that he has met with similar success in other cases. He suggests that in any case where the laceration of the perineum is very extensive, it might probably be advisable to employ two rings instead of one.

*Hannoversche Annalen.* Bd. v. Heft 1.

*On the Treatment of Croup by frequently repeated Emetics.* By Dr. MAROTTE, formerly Interne of the Hospitals of Paris.

A paper on this subject by M. de Larroque, which appeared in the Bulletin Général de Thérapeutique for September, 1840, led the writer to resort to this mode of treatment. The emetic which he employed consisted of the tartrate of antimony in large and frequently repeated doses. He does not, however, attach so much importance to that preparation as to the emetic effect which follows the administration of many other medicines besides tartar emetic. The employment of local depletion, carried so far as to cause some faintness, has appeared to Dr. Marotte to be very beneficial, and to favour the subsequent operation of the emetic. Neither the dose of the medicine, nor the times for its repetition can be regulated by any fixed rule; but the former may require to be increased and the latter to be rendered more frequent, according to the urgency of the symptoms. Vomiting should be kept up until a marked influence is produced on the cough and respiration, and must be repeated as often as the symptoms appear about to return. The fear of exciting gastritis Dr. Marotte treats as groundless; and details three cases in proof of the good results of the emetic plan of treatment, and its freedom from evil consequences.

[In Vol. IX. p. 573 of our Journal will be found an abstract of a paper by Mr. Wilson, on the treatment of croup by large doses of tartar emetic. That gentleman, however, was of opinion that the antimony produced a beneficial effect, independent of its emetic action. In other respects, however, the views of Mr. Wilson closely coincide with those of Dr. Marotte.

*Gazette Médicale. Janvier* 1, 1842.

